# MicroRNAs and Their Big Therapeutic Impacts: Delivery Strategies for Cancer Intervention

**DOI:** 10.3390/cells11152332

**Published:** 2022-07-29

**Authors:** Charles Holjencin, Andrew Jakymiw

**Affiliations:** 1 Department of Oral Health Sciences, James B. Edwards College of Dental Medicine, Medical University of South Carolina (MUSC), Charleston, SC 29425, USA; holjenci@musc.edu; 2 Department of Biochemistry & Molecular Biology, College of Medicine, Hollings Cancer Center, Medical University of South Carolina (MUSC), Charleston, SC 29425, USA

**Keywords:** microRNA, miRNA therapeutics, miRNA delivery, RNA silencing, cancer, cell-penetrating peptides, CPPs

## Abstract

Three decades have passed from the initial discovery of a microRNA (miRNA) in *Caenorhabditis elegans* to our current understanding that miRNAs play essential roles in regulating fundamental physiological processes and that their dysregulation can lead to many human pathologies, including cancer. In effect, restoration of miRNA expression or downregulation of aberrantly expressed miRNAs using miRNA mimics or anti-miRNA inhibitors (anti-miRs/antimiRs), respectively, continues to show therapeutic potential for the treatment of cancer. Although the manipulation of miRNA expression presents a promising therapeutic strategy for cancer treatment, it is predominantly reliant on nucleic acid-based molecules for their application, which introduces an array of hurdles, with respect to in vivo delivery. Because naked nucleic acids are quickly degraded and/or removed from the body, they require delivery vectors that can help overcome the many barriers presented upon their administration into the bloodstream. As such, in this review, we discuss the strengths and weaknesses of the current state-of-the-art delivery systems, encompassing viral- and nonviral-based systems, with a specific focus on nonviral nanotechnology-based miRNA delivery platforms, including lipid-, polymer-, inorganic-, and extracellular vesicle-based delivery strategies. Moreover, we also shed light on peptide carriers as an emerging technology that shows great promise in being a highly efficacious delivery platform for miRNA-based cancer therapeutics.

## 1. Introduction

Since the reported finding of the first human miRNA in 2000 [1] and, subsequently, the discovery of cancer-associated miRNAs several years later [2], miRNAs have come into focus as feasible therapeutic drug candidates and/or therapeutic targets for cancer intervention. With the global incidence of cancer burden and cancer-related deaths rising to 19.3 million and 10 million in 2020, respectively, and due to a predicted ~50% increase in cancer burden worldwide by 2040, according to the most recent GLOBOCAN estimates from the International Agency for Research on Cancer (Global Cancer Observatory; Lyon, France) [3,4], it is evident that continued therapeutic efforts are needed to help combat this devastating disease. Thus, with miRNAs being found to be aberrantly expressed in many cancer types [5,6,7,8,9], it is not surprising that miRNA therapeutics has evolved and developed into a promising approach (via its manipulation of miRNA expression levels) for cancer treatment.

Despite the progress made in the preclinical development of miRNA therapeutics over the past several decades [10,11,12,13], its transition to the clinic still faces significant hurdles. Presently, of the 11 developed miRNA therapeutics that have reached clinical trials (Table 1) [10,12,14,15], four studies have been terminated/discontinued/withdrawn, five have completed phase I or II trials, and two are currently undergoing active phase I or II trials. Notably, though, none have yet reached phase III trials, nor are being apparently further developed and/or advanced for cancer-related treatments. This slow transition to the clinic could be in-part due to the numerous pharmacological challenges confronted by miRNA-based cancer therapies, which can include rapid degradation by ribonucleases in the bloodstream, renal and reticuloendothelial system (RES) clearance, poor penetration into tumor tissues due to mechanical and biological barriers, immunotoxicity, neurotoxicity, cell/tissue-type specific delivery, poor intracellular delivery, and endosomal entrapment, with subsequent degradation in lysosomes [13]. Notwithstanding, continued advancements in RNA chemistry, and in particular, delivery technologies, are enabling miRNA therapies to overcome these limitations, making the application of this form of therapy in the clinic for cancer treatment that much more a possible reality. In this context, the main focus of this review is on the advancements of varied miRNA delivery platforms for cancer intervention, along with a discussion of the emergence of peptides as potential carriers for miRNA-based cancer therapeutics.

## 2. RNA Silencing and miRNAs

RNA silencing is an evolutionary conserved eukaryotic post-transcriptional gene regulatory mechanism by which noncoding RNAs (ncRNAs) induce the sequence-specific inhibition of target gene expression and/or protein synthesis. One particular class of ncRNAs that can induce RNA silencing and affects many cellular processes and developmental pathways are the small (20–25 nucleotides (nt) in length) ncRNAs known as miRNAs [16,17,18,19]. Specifically, miRNAs regulate gene expression by either repressing translation or inducing mRNA degradation, depending on the degree of sequence complementarity with the target mRNAs [16,20]. To date, >60% of human protein-coding genes are predicted to contain miRNA binding sites and the total number of human miRNAs are estimated to be ~2300 [21,22,23,24].

The biogenesis of mature miRNAs initiates from long primary transcripts, termed primary-miRNAs (pri-miRNAs), which harbor hairpin structures comprising the miRNA sequences that are transcribed predominantly by RNA polymerase II, but also by RNA polymerase III [17,20,25]. Many canonical miRNAs are derived from the introns/exons of long ncRNA transcripts, as well as the introns of protein-coding precursor-mRNAs, where these pri-miRNAs can consist of either a single miRNA gene unit or a cluster of often related miRNA genes [17,19,20]. Following transcription, the hairpin structures within the pri-miRNAs are recognized and processed by the nuclear microprocessor complex, comprising an RNase III enzyme, Drosha, in complex with DGCR8, resulting in the liberation of ~70 nt stem-loop precursor miRNAs known as pre-miRNAs [26]. Subsequently, the pre-miRNAs are exported to the cytoplasm by the action of Exportin-5 [27,28,29], where they undergo final processing by another RNase III enzyme, Dicer, to generate ~20 base-pair miRNA duplexes [17,30,31,32]. Afterward, the miRNA duplex is then incorporated into an Argonaute (AGO) protein, where one strand (i.e., the “guide” strand) is selected to become the mature miRNA and its complementary strand (i.e., the “passenger” strand) is discarded, thus forming the mature RNA-induced silencing complex (RISC) [17,19,20,33,34,35]. Strand selection preference is given based on the strand possessing the least stable paired 5′-end [36,37], as well as a 5′-terminal A or U residue [38,39]. Once loaded in the RISC, the mature miRNA subsequently pairs to target sites generally located in the 3′-UTR of mRNAs to direct RNA silencing [16,23,40].

The interactions of miRNAs with their mRNA targets are largely based on their seed sequence, which comprises nucleotides 2–8 (from the 5′-end), and whose sequence similarities are used as a basis for grouping miRNAs into families [16,17]. In addition, other atypical sites on the miRNA, such as the 3′-supplementary site, can further supplement the seed interactions through additional base pairings [16,17,41,42,43]. Thus, the extent of the pairing between the miRNA and its mRNA target will ultimately dictate the mechanism of gene silencing, where very extensive pairings will result in cleavage/slicing of the mRNA target and less extensive pairings will induce translational repression or mRNA decay via deadenylation, decapping, and 5′-3′ exonuclease activity [17,20,44,45]. Although both the translational inhibition and mRNA decay modes of miRNA-mediated repression are thought to be interconnected [20,45], the mechanism of mRNA decay appears to be generally responsible for 66–90% of silencing in cells [17,20,46,47].

## 3. miRNAs, Cancer, and Therapeutic Approaches to miRNA Replacement/Inhibition

miRNAs can form complex, intertwined networks of interactions in their abilities to regulate gene expression [48], as a single miRNA can silence many different mRNAs, and one mRNA can be regulated by multiple miRNAs [20]. Moreover, individual miRNAs and miRNA clusters can regulate entire cellular pathways, with multiple miRNAs also being able to control intermeshed regulatory networks [20,48]. Hence, through this network-regulatory role of miRNAs, combined with miRNAs also having been implicated in regulating practically every cellular process and being essential for animal development, cell differentiation, and homeostasis [17,20,48,49], it is not surprising that alterations in miRNA expression have been associated with different human diseases, including cancer [48,50].

An overwhelming amount of evidence indicates that miRNAs exhibit abnormal expression levels in malignant cells and tissues and that they are deeply involved in tumor onset and progression through their tumor-promoting and/or tumor suppressive behaviors [10,50]. More specifically, the dysregulation of miRNA expression levels in cancer has been associated with a number of biological hallmarks of human cancer development, including sustained proliferative signaling, evasion of anti-growth signals and immune destruction, resistance of apoptosis, activation of invasion/metastasis, induction of angiogenesis, and promotion of genomic instability and inflammation [5,10]. The underlying mechanisms that contribute to the dysregulation of miRNA expression levels in human malignancies are often attributed to the amplification, deletion, or translocation of miRNA genes (i.e., the alteration of genomic miRNA copy numbers or gene locations) [5]. However, the perturbation of other cellular mechanisms can also contribute to their abnormal expression, which can involve changes to their transcriptional or epigenetic control, as well as defects in the miRNA biogenesis machinery [5].

**Figure 1:**
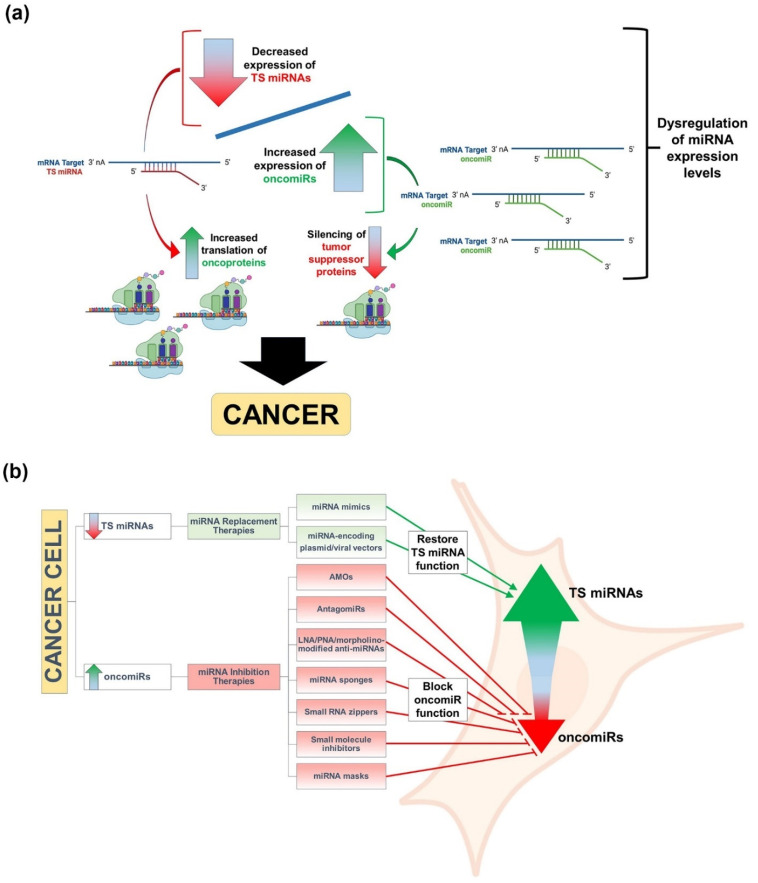
Graphical depictions highlighting the different types of cancer-associated miRNAs and the molecular therapeutic approaches to alleviating their abnormal expression in cancer. (**a**) Classes of tumor-associated miRNAs and their mechanisms of promoting cancer. Tumor-associated miRNAs are classified as either oncogenic miRNAs (oncomiRs) or tumor suppressor miRNAs (TS miRNAs), and the dysregulation of the expression levels of these tumor-associated miRNAs leads to cancer onset and progression. In particular, oncomiRs are typically overexpressed in malignant cells/tissues and promote cancer by silencing mRNAs encoding tumor suppressor proteins (green curved arrow), whereas TS miRNAs are downregulated and result in increases in the translation of their target oncoprotein-encoding mRNAs (red curved arrow). (**b**) miRNA inhibition and replacement therapies for cancer treatment. miRNA inhibition and replacement therapies are molecular interventions designed to either suppress oncomiR or restore TS miRNA functions, respectively. Approaches to enhance TS miRNA function via miRNA replacement therapy have been achieved through the use of chemically synthesized/modified, double-stranded miRNA mimics, as well as through the use of plasmid or viral vectors engineered to encode specific TS miRNAs that can replenish the lost miRNAs within cancer cells. Alternatively, approaches to antagonize oncomiR function via the use of antimiRs, resulting in the impairment of cancer progression, have been achieved through the use of antisense miRNA oligonucleotides (AMOs), antagomiRs, locked nucleic acid (LNA)/peptide nucleic acid (PNA)/morpholino-modified anti-miRNAs, miRNA sponges, small RNA zippers, small-molecule inhibitors, or miRNA masks. Select images within the figure were acquired from BioRender.com.

Based on their modulating effect on the expression of their target genes, tumor-associated miRNAs can be classified into two categories: (1) oncogenic miRNAs (oncomiRs) and (2) tumor suppressor miRNAs (TS miRNAs) [8,10]. In general, oncomiRs are typically found to be overexpressed in malignant cells/tissues and promote cancer by silencing mRNAs encoding tumor suppressor proteins, whereas TS miRNAs are usually downregulated and result in increases in the translation of their target oncogenic mRNAs (Figure 1a) [8,10,50]. Deep sequencing and miRNA profiling studies have provided direct evidence that miRNA expression is dysregulated in cancer and that its varied and unique signatures can be used for tumor classification, diagnosis, and prognosis [5,6,7,9]. More importantly, the dysregulation of miRNAs in cancer has also been harnessed as a potential therapeutic modality, by either miRNA replacement therapy using miRNA mimics or inhibition of miRNA function by antimiRs to restore TS miRNA or suppress oncomiR activities, respectively (Figure 1b) [10,50,51,52,53]. Approaches to enhance TS miRNA activity via miRNA replacement therapy have been achieved through the use of chemically synthesized/modified, double-stranded miRNA mimics, as well as through the use of plasmid or viral vectors engineered to encode specific TS miRNAs that can replenish the lost miRNAs within the cancer cells, thus inducing silencing of the targeted oncogenic mRNAs and impairing tumor progression [54,55,56,57,58]. Alternatively, approaches to antagonize oncomiR activity via the use of antimiRs capable of restoring tumor suppressor protein expression and halting cancer progression have been achieved through the use of antisense miRNA oligonucleotides (AMOs), antagomiRs, locked nucleic acid (LNA)/peptide nucleic acid/morpholino-modified anti-miRNAs, miRNA sponges, small RNA zippers, small-molecule inhibitors, or miRNA masks [59,60,61,62,63,64,65,66,67,68,69]. Nonetheless, although advances have been made in the abovementioned strategies in circumventing the abnormal expression of miRNAs in cancer cells, the intracellular delivery of these therapeutic miRNA mimics and antimiRs still presents a challenge to the development of effective miRNA-based cancer therapeutics, especially in vivo, that must be overcome in order to make them clinically relevant. Fortunately, there has been and continues to be much progress made in the development of highly efficacious delivery systems that are making miRNA therapeutics a clinical reality for cancer intervention.

## 4. Delivery Platforms for miRNA-Based Cancer Therapeutics

**Figure 2:**
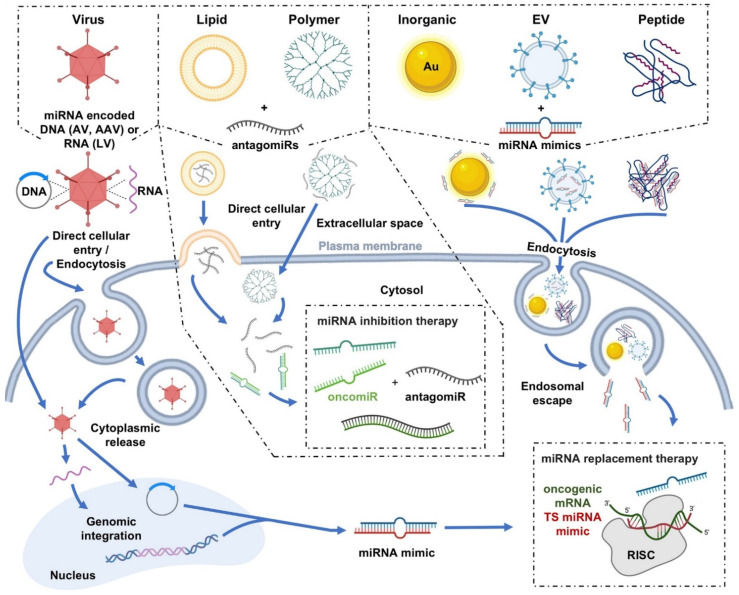
Graphical depictions highlighting the varied miRNA delivery platforms developed for cancer therapeutics, as well as their mechanisms of cellular internalization. Schematic diagram illustrating the generalized modes of cell entry for both viral and nonviral miRNA delivery systems. To cross the plasma membrane of the targeted cell, many of the delivery systems utilize multiple different cellular entry routes, but in general, utilize either direct cellular entry mechanisms or endocytosis-based uptake pathways [70,71]. For example, viral vectors, such as adenovirus (AV), adeno-associated virus (AAV), and lentivirus (LV), can utilize either direct entry mechanisms or endocytosis-based uptake pathways in their delivery of miRNA mimic-encoded RNA/DNA cargo into cells. Other examples, in terms of nonviral delivery systems, can include direct cytoplasmic entry via lipid fusion of a lipid-based vector with the plasma membrane or direct cellular entry of a polymer (e.g., polyamidoamine (PAMAM))-based vector in the delivery of antagomiR cargo. Additionally depicted are examples of cellular internalizations via endocytosis of inorganic (e.g. gold (Au)), extracellular vesicle (EV), and peptide-based vectors in complex with miRNA mimic cargo. Select images within the figure were acquired from BioRender.com.

Some of the most significant barriers to the widespread use of miRNA replacement/inhibition therapies for the treatment of cancer are consequences of systemic delivery and limitations in tumor-tissue specificity. Thus, because the therapeutic manipulation of miRNA expression is predominantly reliant on nucleic acid-based molecules, delivery vectors are needed to help them overcome not only the systemic barriers, such as rapid renal clearance, but also clinical barriers, such as biosafety [13]. The following section highlights the current state-of-the-art viral and nonviral delivery systems that have been demonstrated to deliver miRNA-based cancer therapies in vitro and in vivo, with a focus on nonviral nanotechnology-based miRNA delivery systems. The graphical depictions of these varied delivery systems, including their generalized modes of cell entry, as well as their strengths and weaknesses, can be found in Figure 2 and Table 2 below.

### 4.1. Viral Delivery

Virus-mediated delivery of miRNAs has been shown to be highly efficacious, where viral vectors can be designed to deliver miRNAs at different stages of biogenesis (i.e., pri-miRNAs and pre-miRNAs). Driven by a viral promotor, pri/pre-miRNA cloned within a plasmid can be transcribed and further processed to the mature miRNA form, enabling it to subsequently act on the target mRNA [57]. Adenoviruses (AdVs), particularly oncolytic AdVs (OAdVs), have been successful in the delivery of antimiRs and miRNA mimics. In fact, OAdV-mediated delivery of antimiRs in the form of long ncRNAs (lncRNAs), which has the therapeutic design advantage of targeting multiple copies of the same miRNA or different miRNAs within a single lncRNA molecule, has been shown to inhibit tumor growth in xenograft murine models of triple-negative breast cancer (TNBC) by simultaneously suppressing oncomiR levels of miR-9-5p, miR-10b-5p, miR-21-5p, miR-23a-3p, miR-29a-3p, miR-155-5p, miR-222-3p, miR-301a-3p, and miR-373-3p [72] and to decrease sorafenib resistance in sorafenib-resistant hepatocellular carcinoma (HCC) by concomitantly targeting miR-21, miR-153, miR-216a, miR-217, miR-494, and miR-10a-5p [73]. Virus-mediated overexpression of miRNAs can also have its advantages by suppressing particular oncogenes, as it has been shown that overexpression of miR-143 can inhibit cell growth and induce apoptosis by targeting KRAS in human colorectal cancer, in vitro [74]. Moreover, virus-mediated overexpression of miR-199 and miR-34a has been found to lead to control of tumor growth by targeting mTOR, c-MET, HIF-1α, and CD44, as well as complete tumor regression by targeting Bcl-2 and SIRT1 in xenograft murine models of HCC [75,76], respectively. Similarly to AdV, adeno-associated virus (AAV)-mediated delivery systems have also had success in the treatment of HCC, as overexpression of miR-342-3p and miR-26a has each demonstrated anti-tumor effects in murine models [77,78] by targeting MCT1 and cyclins D2 and E2, respectively. Additionally, Bhere and colleagues recently showed that AAV-mediated delivery of anti-miR-21 and miR-7 resulted in decreased cell proliferation, migration, and invasion of human prostate and colon cancer cells, in vitro, and a significant reduction in tumor burden in glioblastoma murine models [79] through targeting of the PI3K/AKT and JAK/STAT3 signaling pathways (via anti-miR-21) and down-regulation of EGFR and p-AKT (via miR-7). Lentivirus-mediated delivery of miR-15a and miR-16 in murine models of chronic lymphoid leukemia [80] and a miRNA sponge targeting miR-494 in murine models of breast cancer [81] have also resulted in beneficial therapeutic effects; however, concerns of lentiviral integration into the host genome have limited their clinical application as a delivery vector.

### 4.2. Nonviral Delivery

Despite being highly efficacious, virus-mediated miRNA-based therapeutic delivery platforms lack clinical desirability due to a number of biosafety concerns, including viral immunogenicity. Although classically thought of as inefficient compared to viral vectors, recent advancements in nonviral delivery platforms, however, are paving the way for nucleic acid-based therapies that make their application that much more feasible in the clinic [13]. As such, the following sections highlight and discuss the pros/cons of the varied nonviral delivery technologies that have been developed for miRNA-based cancer therapeutics, including polymer nanoparticles, lipid-based nanoparticles, inorganic nanoparticles, extracellular vesicles, and an emerging technology, which is of particular relevance to our own studies—peptide carriers.

#### 4.2.1. Polymer Nanoparticles

Polymeric delivery systems have found success as suitable vectors for delivery of nucleic acids due to their high stability and flexibility, and the facile ability to make substitutions and/or additions of functional groups [82]. In fact, one particular polymer, poly lactic-co-gycolic acid, has gained FDA approval as a delivery vector and is in phase II clinical trials for the delivery of therapeutic small interfering RNA (siRNA) molecules (NCT01676259) [83]. The ability to control the molecular weight, polymer composition, and architecture of polymers allows for the manipulation of size, morphology, charge, pKa, membrane interactions, and biodegradability [84]. Moreover, polymer-based vectors are composed of a variety of materials, including natural polymers, such as collagen, gelatin, and chitosan, synthetic polymers, and combinations of natural and synthetic polymers. The following subsections pertaining to polymer nanoparticles detail polymer-based vectors that have found success in the delivery of specific miRNA-based cancer therapies.

##### Polyethylenimines

Polyethylenimines (PEI) are second-generation cationic polymers that are frequently utilized for therapeutic gene delivery [84]. PEI is composed of many positively charged amino groups, allowing for complexation with anionic RNA molecules and shielding from degradation, as well as enabling the proton sponge effect, which promotes escape from endosomes after endocytosis [85]. Indeed, PEI has been shown to be an effective delivery vector of miRNA for treatment of various cancers, including miR-33a and miR-145 for the treatment of colon cancer [86] and miR-708-5p mimics for the treatment of metastatic non-small cell lung cancer [87]. Although successful, PEI alone is not as desirable as other delivery vectors, due to the excess positive charge and low degradability due to the binding of serum proteins [57]. As such, PEI in combination/conjugation with other lipids and polymers has been investigated to mitigate these undesirable effects and has found some success. For example, PEI-polyethylene glycol (PEG)-mediated delivery of miR-34a and miR-150 has been shown to be effective for treatment of HCC [88] and to effectively reduce the cell viability of chronic myeloid leukemia cells [89], respectively. Additionally, PEI-hyaluronic acid (HA)-PEG-mediated delivery of a plasmid encoding miR-125b has been demonstrated to inhibit tumor growth and induce apoptosis in a murine lung cancer model [90]. Likewise, polyurethane-PEI-mediated delivery of a plasmid encoding miR-145 has shown success for the treatment of glioblastoma [91], and PEI-antagomiR-126 complexes, loaded into liposomes, have been demonstrated to effectively target leukemic stem cells in vivo for the treatment of acute myeloid leukemia [92].

##### Polyamidoamine

Polyamidoamine (PAMAM) is a hyperbranched synthetic polymer that is positively charged and biodegradable. While the overall positive charge of the polymer allows for complexation with nucleic acids, it promotes hepatic accumulation and toxicity [93]. Consequently, PAMAM is frequently found in combination/conjugation with other lipids and polymers. For instance, PAMAM-PEG-mediated delivery of a miR-34a-expressing plasmid has been shown to have anti-tumor effects in non-small cell lung cancer [94], and nanographene oxide (NGO)-PEG-PAMAM-mediated delivery of anti-miR-21 has proven effective in reducing migration and invasion of non-small-cell lung cancer A549 cells, in vitro [95].

##### Chitosan

Chitosan is a biocompatible, natural polysaccharide. It is a deacetylated derivative of chitin, which is found in the exoskeleton of insects, crustaceans, and fungi, making it the second most abundant natural polymer [96]. Chitosan consists of repeating units of β-1,4 linked N-acetyl-D-glucosamine and D-glucosamine [97], and has been described as having a profound binding affinity for miRNAs [10]. Chitosan has been shown to be an effective delivery vector in the treatment of breast cancer, through the complexation of miR-200c [98,99], and in the treatment of prostate bone metastasis, through the complexation of miR-34a mimics [100].

##### Poly Lactic-Co-Gycolic Acid

As previously mentioned, poly lactic-co-gycolic acid (PLGA) is an FDA-approved polymeric delivery vector. These polymers are polyesters and are negatively charged, biodegradable, and biocompatible. PLGA is also hydrophobic, which is thought to impair its miRNA delivery efficacy [101]. As a result, PLGA in combination/conjugation with various lipids and polymers, both synthetic and natural, have been investigated, with some combinations proving effective in mediating miRNA delivery for the treatment of various cancers. In particular, PLGA-chitosan complexes containing miR-34a mimics have been shown to inhibit tumor growth of multiple myeloma xenografts and resulted in the greater survival of treated NOD-SCID tumor-bearing mice [102]. Additionally, PLGA-HA-PEI in complex with a miR-145-encoding plasmid was shown to reduce tumor growth in a murine xenograft model of colon cancer [58], and PLGA-PEG-anti-miR-21, PLGA-PEG-anti-miR-10b/21, and PLGA-PEG-miR-122 complexes were demonstrated to be effective in treating HCC [103], breast cancer [104,105], and colon cancer [106], respectively.

#### 4.2.2. Lipid-Based Nanoparticles

The ease of use and versatility of lipid-based nanoparticles in the form of liposomes have made them the most widely used nanoparticle for the delivery of nucleic acid-based therapies, which includes miRNAs. Liposomes are spherical structures with a hydrophilic core that is separated from the external environment by a phospholipid bilayer. Liposomes can accommodate hydrophobic molecules within the bilayer, hydrophilic molecules within the liposome core, and amphiphilic molecules at the interphase between the bilayer and core [107]. Due to their phospholipid composition, liposomes can interact with cell membranes, which leads to efficient delivery of cargo.

##### Cationic Liposomes

The first generation of liposomes were cationic in nature, which allowed for electrostatic interactions with nucleic acid-based cargos, as well as with the negatively charged surfaces of cells [108,109]. While advantageous for drug loading and delivery, this positive-charge property, however, was found to limit the cell specificity of cationic liposomes, allowed interactions with serum proteins, and increased the susceptibility of uptake by RES [110,111]. Despite these challenges within the circulatory system, a significant tumor reduction in a xenograft tumor mouse model of colorectal cancer was still observed through cationic liposome-based nanoparticles loaded with miR-139-5p mimics, albeit with the liposomes also possessing other functionalized moieties [112]. Decreased levels of miR-143 and miR-145, which are associated with colorectal carcinoma, and the delivery of miR-143 and miR-145 mimics using cationic liposomes to restore their levels have also been shown to reduce cell proliferation in a number of colorectal cancer cell lines [113]. In addition to colorectal cancer, cationic liposome-mediated delivery of a miR-7-encoding plasmid and miR-29 mimics have been demonstrated to significantly reduce tumor sizes in xenograft tumor mouse models of lung cancer [114,115].

##### Neutral Liposomes

To reduce the charge-associated shortcomings of cationic liposomes, neutral liposomes were developed by the inclusion of helper lipids, such as 1,2-dioleoyl-sn-glycero-3-phosphoethanolamine (DOPE) [116], PEG [117], phosphatidylcholines (PCs) [118], and cholesterol [119]. These modifications have led to reduced RES uptake, which allows for increased half-life of the neutral liposomes within the bloodstream [110,111]. Taking advantage of the helper lipid phosphocholine, an intermediate of PC, Trang and colleagues found that neutral liposome-mediated delivery of miR-34a and let-7b mimics resulted in the significant reduction in tumor burden in a K-Ras-activated autochthonous mouse model of non-small cell lung cancer (NSCLC) [120].

##### Ionizable Liposomes

Further optimization of lipid-based nanoparticles has resulted in the generation of ionizable liposomes, which are cationic at low pH and neutral/anionic at neutral or higher pH levels. The ability to change charge states with respect to extracellular pH gives ionizable liposomes enhanced cell selectively characteristics that makes them more clinically translatable. In fact, an ionizable liposome-miRNA complex (comprising miR-34 mimics; MRX34) made it as far as phase I clinical trials for treatment of liver cancer and metastasis (NCT01829971; Table 1) [121,122]. However, this trial was stopped due to severe immune-related adverse events, which resulted in the death of four patients [122]. Despite this unfortunate setback, another group has shown that ionizable liposomes could still hold promise as delivery vehicles for miRNA therapeutics, as they have found, at least at the preclinical level, that ionizable liposome-mediated delivery of miR-200c mimics could result in enhanced radiosensitivity in a xenograft mouse model of lung cancer [123]. Additionally, another study found that delivery of a miR-199b-5p mimic using an ionizable cationic liposome [15] could significantly impair Hes-1 (a downstream effector of the canonical Notch and noncanonical SHH pathways) and cancer stem cell markers in a number of different tumorigenic cell lines, including colon (HT-29, CaCo-2, and SW480), breast (MDA-MB231T and MCF-7), prostate (PC-3), glioblastoma (U-87), and medulloblastoma (Daoy, ONS-76, and UW-228) cancer cell lines [124].

#### 4.2.3. Inorganic Nanoparticles

Inorganic nanoparticles are desirable as delivery vectors because they can be designed to be biocompatible, nonimmunogenic, and nontoxic, and the size, shape, and porosity of particles can be controlled [10,125]. Nevertheless, the use of inorganic materials for delivery of miRNAs still faces challenges, such as protection from degradation in vivo, as well as endosomal escape [13]. The following section describes examples of common inorganic vectors utilized for delivery of miRNAs, however, the examples discussed herein are not a complete representation of all developed technologies.

##### Calcium Phosphate

Calcium phosphate (CaP) nanoparticles, composed of hydroxyapatite [Ca_5_(PO_4_)_3_OH], the inorganic component of bone and teeth, are described as being the most successful inorganic vectors for miRNA therapeutics [10], particularly for the treatment of colon/colorectal cancers. CaP owes this success to its unique in vivo characteristics, including its biocompatibility and biodegradability properties. Moreover, CaP’s susceptibility to acidic conditions allows for endocytic escape, where once these CaP nanoparticles are endocytosed, the acidic environment of the endosome dissolves them, resulting in subsequent increases in ionic strength that lead to osmotic swelling and the release of cargo [126]. As previously mentioned, CaP nanoparticle-mediated delivery of miRNAs has been particularly successful in the treatment of colon/colorectal cancers, as delivery of miR-4711-5p [127], miR-4689 [128], and miR-29b [129] mimics were found to effectively inhibit tumor growth in xenograft colon/colorectal cancer mouse models.

##### Silica

Silica-based nanoparticle technologies are desirable due to their biocompatibility, large surface area, well-defined chemical properties, and ability to control characteristics, such as pore structure [130]. One type of silica-based nanoparticle that has found success with miRNA delivery is the mesoporous silica nanoparticle (MSNP). MSNPs have a/an: (i) tunable particle size, which is important for endocytosis; (ii) stable and rigid framework, making them more resistant to heat, pH, mechanical stress, and hydrolysis-mediated degradation; (iii) uniform and tunable pore size, allowing for controlled drug loading; (iv) high surface area (>900 m^2^/g) and large pore volume (>0.9 cm^3^/g), which allows for increased drug loading; (v) interior and exterior surface, permitting selective functionalization of either surface; (vi) unique “honeycomb-like” porous structure, which aids in decreased premature drug release or leaking [131]. Taking advantage of these MSNP properties, Bertucci and colleagues successfully induced apoptosis in temozolomide (TMZ)-resistant T98G glioblastoma cells, in vitro, by loading the MSNPs with the anti-cancer drug TMZ and decorating them on the surface with a polyarginine-peptide nucleic acid (R8-PNA) antimiR conjugate designed to target miR-221, a miRNA, whose downregulation was previously reported to sensitize glioma cells to TMZ [132,133].

##### Gold

Gold (Au)-based nanoparticles (AuNPs) are well suited for delivery of nucleic acids, particularly after the addition of various functional groups. AuNPs have multifunctional monolayers, allowing for the addition of multiple functional moieties, which can control cytotoxicity, biodistribution, and excretion [134,135,136,137,138,139]. AuNPs can also be easily scaled with low size dispersity [140]. Due to these characteristics, AuNPs have found success in delivery of miRNAs for the treatment of various cancers. In particular, miR-375 mimic-coated AuNPs were observed to reduce tumor volume in primary and xenograft tumor mouse models of HCC [141]. Additionally, AuNPs formulated with PEG were found to mediate the highly efficient cell uptake of miRNAs and could decrease cell proliferation upon delivery of a miR-31 mimic into neuroblastoma (NGP and SH-SY5Y) and ovarian (OVCAR8 and HEYA8) cancer cell lines [142]. Moreover, Gilam and colleagues showed that in combination with the chemotherapy drug, cisplatin, AuNPs functionalized with PEG and a tumor-homing peptide, embedded within a hydrogel, could mediate the efficient local, selective, and sustained release of co-complexed miR-96 or miR-182 mimics, leading to the reduction in primary tumor size and metastasis in a breast cancer mouse model [143].

#### 4.2.4. Extracellular Vesicles

Extracellular vesicles (EVs) are cell-derived nanovesicles that transport DNA, RNA, proteins, and lipids for cellular communication and activation of signaling pathways [144]. While EVs transport mRNA and other RNA species, such as lncRNAs and circular RNAs, miRNAs are perhaps the most abundant cargo molecule in EVs, particularly in exosomes [145]. In fact, these exosome-associated miRNAs have significant roles in the post-transcriptional regulation of gene expression and participate in the mediation of inflammatory reactions, cell migration, proliferation, apoptosis, autophagy, and epithelial-mesenchymal transition [145]. It, therefore, stands to reason that exploiting EVs for therapeutic miRNA delivery has its advantages over other delivery vectors. As a natural biomolecular carrier possessing specific ligands, EVs can be selectively delivered to cell types bearing specific surface receptors [146]. Additionally, EV ligands, such as CD47, can aid in their protection from phagocytes [147]. Moreover, lipid bilayer-encapsulated miRNA cargo is protected from RNase-mediated degradation, as well as from other circulatory system obstacles. This lipid bilayer also allows for direct fusion of the EV with the target cell membrane, with the subsequent release of cargo directly into the cytoplasm of the target cell, thus evading potential endosomal entrapment of the cargo; however, it should be noted that EVs can also undergo receptor-mediated endocytosis [148,149,150]. Though a relatively new delivery platform for therapeutic nucleic acid-based drugs, EV-mediated delivery of miRNAs has already shown promising therapeutic responses in various cancers. For example, EV-mediated delivery of a chemically modified miR-143 mimic, a plasmid expressing miR-146b, and a miR-145 mimic has each been observed to have therapeutic effects in colon cancer [151], glioma [152], and lung cancer [153], respectively. Other therapeutic uses of EVs have also been reported in the delivery of a plasmid expressing miR-122 in HCC [154] and a chemically synthesized miR-199a-3p mimic in ovarian cancer [155]. Similar to nonviral vectors discussed thus far, exosomes also have the advantage of being modified to contain different functional surface moieties. One interesting example of this type of modification was reported by Ohno and colleagues, where they engineered exosomes to express an epidermal growth factor receptor (EGFR)-specific targeting peptide, GE11, on their surfaces, which were then subsequently used to target a TS miRNA to EGFR-expressing breast cancer cells [156]. In particular, when GE11-positive exosomes containing the TS miRNA, let-7a, were administered to EGFR-expressing breast cancer xenograft tumor-bearing mice, these GE11-positive let-7a-loaded exosomes were observed to not only target the tumors, but also impair their development [156].

#### 4.2.5. Peptides

**Figure 3:**
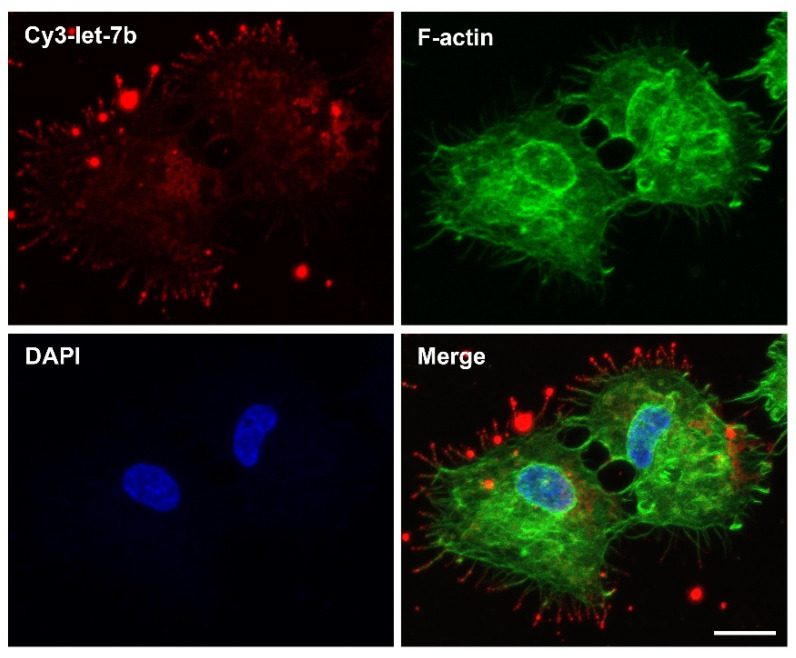
RD3AD peptide-mediated localization of complexed let-7b miRNAs to filopodia and delivery into cancer cells. Confocal fluorescence microscopy analysis of CAL 27 oral cancer cells 2 h post-treatment with a synthetic Cy3-labeled let-7b miRNA duplex (Cy3-let-7b; red) in complex with the RD3AD peptide. Filopodia (green) were stained with the F-actin label Alex Fluor 488 phalloidin, and nuclei (blue) were counterstained with DAPI. The merged images are also presented. Scale bar: 15 µm. For details on materials and methods used, please see Supplementary Materials.

The use of peptides as a delivery vector of nucleic acid-based therapeutics was initially described over two decades ago [157,158,159], but has only recently begun to gain popularity. Peptides are favorable delivery vectors because of the diversity of their physiochemical properties and functions [160]. The controlled ability to modify their amino acid sequences and ease of synthesis allows for the production of peptides that can overcome many of the systemic circulation-associated barriers faced by nucleic acid-based therapies. As such, many different classes of peptide carriers exist, one of which, the cell-penetrating peptides (CPPs), are proving to be highly efficacious and clinically translatable for the treatment of various cancers, as suggested by their presence in phase I and II clinical trials [161,162,163,164,165], as either a therapeutic agent alone or as a delivery agent for macromolecular therapeutics [166]. CPPs are typically 4–40 amino acid residues in length [167], can penetrate the plasma membrane of a cell and facilitate the delivery of different cargos [168], and are considered by some to be the most promising nonviral delivery platform for improvement of intracellular trafficking of nucleic acid-based cargos [169], which have included DNA, RNA, siRNA [170], and more recently, even those associated with miRNA-based therapeutics. For instance, one particular peptide carrier, named FA-R9-FP_cas3_, comprising a folate receptor-targeting ligand, folic acid (FA), a nona-arginine CPP (R9), and a Caspase-3-sensitive imaging probe (FP_cas3_), was used to form a multi-functional peptide-miRNA nanocomplex consisting of the miR-34a mimic that was capable of suppressing tumor growth upon tail vein injection into living mice bearing subcutaneous HeLa tumors [171]. Moreover, because molecular imaging is such a powerful tool for visualization and quantification of pathological processes, such as cancer, Yang and colleagues recently demonstrated that a CPP, PepFect6, could also be used in complex with a radioactively-labeled AMO designed to target the oncomiR, miR-21, to successfully image miR-21 expression in A549 lung adenocarcinoma xenografts, thus demonstrating a promising method for the noninvasive imaging of miRNA expression levels in vivo [172]. Additionally, although not examples of cancer-related applications, peptides such as LMWP and PepFect6 have also been shown to successfully deliver miRNA mimics, including miR-29b, to stem cells to promote osteoblastic differentiation [173] and miR-146a (a known anti-inflammatory miRNA) to inhibit inflammatory responses in a murine model of irritant contact dermatitis [174], respectively. Lastly, regarding our own studies, we have also reported that CPPs can be effective carriers of therapeutic small ncRNAs, particularly siRNAs, which are similar to miRNAs in function in that they both can induce post-transcriptional gene silencing, but differ in that siRNAs typically inhibit the expression of a single mRNA target, whereas miRNAs normally regulate the expression of multiple mRNA targets [43]. More specifically, we have demonstrated that a CPP, named 599, could enhance the intracellular delivery and bioavailability of siRNAs in oral cancer cells in vitro, as well as induce oncogene silencing upon intratumoral administration, resulting in significant inhibition of tumor growth in an orthotopic oral cancer mouse model [54,175]. In subsequent work, we also demonstrated that co-complexation of 599 with a cancer cell-targeting peptide could synergistically mediate the effective targeting/delivery of siRNAs to xenograft oral cancer tumors upon systemic administration and significantly enhance silencing of the targeted oncogene [176]. More recently, in an effort to improve upon the efficacy of siRNA uptake and gene silencing mediated by 599, we found that a 599 peptide variant, RD3AD, which exhibited enhanced siRNA uptake and gene silencing in comparison to 599, also directed siRNAs to specific cell-surface protrusions, identified as filopodia [177]. Intriguingly, filopodia are highly dynamic, elongated, and thin cellular processes that have been reported to facilitate the highly efficient cell entry of viruses, bacteria, activated receptors, lipo/polyplexes, and exosomes by mediating their retrograde transport and/or “surfing” along the structures toward the cell body [178,179,180,181,182], where, at the filopodial base, endocytic hotspots potentially allow for easier cell entry [179]. Of particular relevance regarding exosomes, which are known transporters of miRNAs [183], is that they can utilize filopodia to “surf” toward endocytic hotspots at the filopodial base, internalize, and then traffic within endosomes to the ER [178], which coincidentally is the central nucleation site of siRNA and miRNA-mediated RNA silencing [184,185]. Hence, one can envision how the targeting of filopodia and the subsequent directed transport of RD3AD-siRNA/miRNA complexes from the filopodia to the ER would potentially allow for a very efficient trafficking route of exogenous siRNAs/miRNAs into the cellular RNA silencing machinery. In fact, recent preliminary data from our lab have found that complexation of RD3AD with a synthetic fluorescently-labeled let-7b miRNA duplex could similarly localize the miRNA mimic to filopodia and direct its entry into cancer cells (Figure 3). The significance is that one can, thus, potentially exploit filopodia-directed cell-entry machineries and subcellular-trafficking routes via CPPs for the development of more effective miRNA therapeutics.

## 5. Conclusions

Despite the pre-clinical promises of miRNA-based cancer therapeutics, the clinical advances of this form of therapy for human cancer intervention have been unfortunately very limited. Of the varied nonviral delivery platforms discussed earlier, only the lipid-based miRNA delivery system, MRX34, had reached Phase I clinical trials (NCT01829971; Table 1) [121,122], but had to be prematurely terminated, as a result of it inducing severe immune-related adverse events that led to a number of patient deaths [122]. Even unconventional miRNA delivery technologies, such as the bacterially-derived nanocell-delivery system (i.e., the EnGeneIC Delivery Vehicle) that was adapted to deliver miR-16 mimics for the treatment of mesothelioma and non-small cell lung cancer and had shown promise after the completion of a Phase I clinical trial (NCT02369198; Table 1) [186,187,188], has had no new updates regarding its progression to Phase II clinical trials since 2017. Nevertheless, although challenges persist in the development of effective delivery vehicles for miRNA-based cancer therapies, the hope remains that with the advances being made in delivery platforms for miRNA therapeutics in conferring stability to the miRNA-associated drug candidate, enhancing cancer cell-specific targeting, and promoting more efficient intracellular delivery of the therapeutic cargo, while limiting potential toxicities and adverse immune responses, it is feasible that this form of cancer therapy will become a clinical reality in the near future. With the advent of nonviral delivery technologies, such as peptide carriers, particularly CPPs, which can be tailored to target specific cancer cells and designed to enhance the cell entry of their associated drug cargo, for example, through the potential exploitation of filopodia-directed cell-entry machineries for improved drug efficacy, it is not unreasonable to assume that through further studies focused on the mechanisms of cell entry of the varied drug delivery systems and subcellular trafficking fates of the delivered nucleic acid-based cargos, that miRNA-based cancer therapeutics will soon have “big” clinical impacts.

## Figures and Tables

**Table 1:** Status of miRNA therapeutics-based clinical trials in USA.

Drug	miRNA Therapeutic	Delivery System	Disease	Status *	Manufacturer
Lademirsen (RG-012/SAR339375)	anti-miR-21	AMO ^a^ inhibitor	Alport Syndrome	Phase II (active) NCT02855268	Regulus Therapeutics; Genzyme
AZD4076/RG-125	anti-miR-103/107	GalNAc-conjugated antimiR	Type 2 diabetes; nonalcoholic fatty liver diseases	Phase I (active) NCT02612662; Phase I/IIa (completed) NCT02826525	AstraZeneca; Regulus Therapeutics
Remlarsen (MRG-201)	miR-29 mimic	Cholesterol-conjugated miRNA duplex	Keloid	Phase II (completed) NCT03601052	miRagen Therapeutics
Cobomarsen (MRG-106)	anti-miR-155	LNA-modified antimiR	Lymphomas; Leukemias	Phase I (completed) NCT02580552; Phase II (terminated) NCT03837457 NCT03713320	miRagen Therapeutics
MRG-110	anti-miR-92a	LNA-modified antimiR	Skin wound	Phase I (completed) NCT03603431	miRagen Therapeutics
MesomiR-1/ TargomiR	miR-16 mimic	EDVs-nonliving bacterial minicells	Mesothelioma; non-small cell lung cancer	Phase I (completed) NCT02369198	EnGeneIC; Asbestos Diseases Research Foundation
Miravirsen	anti-miR-122	LNA-modified antimiR	Hepatitis C	Phase I and II (completed) NCT01646489 NCT01200420 NCT01872936 NCT02031133 NCT02508090	Santaris Pharma A/S; Hoffmann-La Roche
RGLS4326	anti-miR-17	AMO inhibitor	Polycystic kidney disease	Phase I (completed) NCT04536688	Regulus Therapeutics
RG-101	anti-miR-122	GalNAc-conjugated antimiR	Hepatitis C	Phase I and II (discontinued)	Regulus Therapeutics
MRX34	miR-34 mimic	Lipid-based nanoparticle (liposome)	Cancer	Phase I (terminated) NCT01829971 NCT02862145	Mirna Therapeutics
pSil-miR200c and PMIS miR200a	Plasmid DNAs encoding miR-200c and a miRNA inhibitor targeting miR-200a	Biodegradable sponge	Tooth extraction	Phase I (withdrawn) NCT02579187	University of Iowa

* ClinicalTrials.gov government identifier numbers are listed for each miRNA therapeutic, if applicable. ^a^ Abbreviations: AMO, antisense miRNA oligonucleotide; GalNAc, N-acteyl-D-galactosamine; LNA, locked nucleic acid; EDVs, EnGeneIC delivery vehicles.

**Table 2:** The highlighted advantages and disadvantages of both viral and nonviral delivery systems in their transport of miRNA-based therapeutics for the treatment of cancer.

miRNA Delivery Platforms for Cancer Therapeutics						
Advantages	Viral	Nonviral				
	Virus	Lipid	Polymer	Inorganic	EV	Peptide
	• Highly efficacious gene delivery	• Non- immunogenic • Control of size, lipid composition and functional groups, and drug loading • Co-delivery of multiple drugs	• Non- immunogenic • Control of size, polymer composition and functional groups, and drug loading • Co-delivery of multiple drugs	• Non- immunogenic • Ease of production • Control of size, composition and functional groups, and drug loading	• Non- immunogenic • Control of functional groups and drug loading • Co-delivery of multiple drugs • Tissue/organ- specific delivery	• Ease/cost of production • Control of physiochemical properties and functions • Tissue/organ- specific delivery
Disadvantages	• Immunogenic • Biosafety concerns	• Non-specific delivery • Low in vivo efficacy • Cytotoxicity	• Non-specific delivery • Low in vivo efficacy • Cytotoxicity	• Low in vivo efficacy	• Lack of experimental data/studies • Inherent diverse composition of EV cargos • Cost of production	• Lack of experimental data/studies

## Supplementary Materials

The following supporting information can be downloaded at: https://www.mdpi.com/article/10.3390/cells11152332/s1, Materials and Methods.
