# Supplementary material for: MicroRNAs and Their Big Therapeutic Impacts: Delivery Strategies for Cancer Intervention
Source: Cells. 2022 Jul 29;11(15):2332. doi: 10.3390/cells11152332 (PMC9367537; doi:10.3390/cells11152332)
Supplement: Supplementary file 1 [file cells-11-02332-s001.zip › cells-1746010-supplementary.pdf]

## Materials and Methods

*Confocal fluorescence microscopy analysis of peptide-mediated miRNA cell uptake*

CAL 27 oral cancer cells (American Type Culture Collection, Manassas, VA, USA), grown to a confluency of 60% on BioCoat Collagen Type I coated 8-chamber slides (Corning, Corning, NY, USA), were rinsed three times with Opti-MEM (Thermo Fisher Scientific, Waltham, MA, USA). Meanwhile, 25 pmol of a chemically synthesized Cy3-labeled let-7b miRNA duplex (Dharmacon, Lafayette, CO, USA) was complexed with RD3AD peptide [177] at a 11.25:1 nitrogen (peptide):phosphate (miRNA) molar ratio for 25 minutes at RT in Opti-MEM. Afterwards, the complexes were added to the cells for incubation at 37°C with 5% $CO_2$ for 2 hours. Subsequently, the cells were rinsed with PBS, fixed in 4% paraformaldehyde at RT for 10 minutes, permeabilized with 0.1% Triton X-100 at RT for 5 minutes, incubated with Alexa Fluor 488 phalloidin (Thermo Fisher Scientific, Waltham, MA, USA) for 20 minutes, and then mounted with a coverslip using VECTASHIELD Mounting Medium with 4',6-diamidino-2-phenylindole (DAPI; Vector Laboratories, Burlingame, CA, USA). Fluorescence images were obtained using a Zeiss (Thornwood, NY, USA) 880 LSM NLO confocal microscope equipped with a 63× objective.
